# The immunosenescence-related gene Zizimin2 is associated with early bone marrow B cell development and marginal zone B cell formation

**DOI:** 10.1186/s12979-015-0028-x

**Published:** 2015-02-22

**Authors:** Takenori Matsuda, Shougo Yanase, Akinori Takaoka, Mitsuo Maruyama

**Affiliations:** Department of Mechanism of Aging, Research Institute, National Center for Geriatrics and Gerontology, 7-430 Morioka, Obu, Aichi 474-8511 Japan; Division of Signaling in Cancer and Immunology, Institute for Genetic Medicine, Hokkaido University, Kita15 Nishi7, Kita-ku, Sapporo 060-0815 Japan

**Keywords:** Zizimin2, Dock11, Bone marrow B cell, Marginal zone B cell

## Abstract

We originally cloned and identified murine Zizimin2 (Ziz2, Dock11) as a guanine nucleotide exchange factor (GEF) for Cdc42 and demonstrated that it activated the formation of filopodia. Since its expression pattern is restricted in immune tissues and Rho GTPases such as Cdc42 function in B cell development and immune responses, we expected Ziz2 to also be associated with B cell development and immune responses. However, the function of Ziz2 has not yet been fully examined *in vivo*. We also recently discovered that Ziz2 expression levels in immune tissues were reduced with aging in the mouse, suggesting that its expression is also associated with the mechanisms of immuno-senescence.

To gain insights into the mechanisms underlying immuno-senescence, we generated Ziz2 knock out (KO) mice and examined the functions of Ziz2 in B cell development and immune responses. We also obtained Zizimin3 (Ziz3; Dock10) KO mice and examined the functions of Ziz3.

The results revealed that Ziz2 KO mice had a higher percentage of early bone marrow B cells (Fraction A), but a reduced fraction of marginal zone (MZ) B cells. In addition, an examination of B cell-specific Ziz2 KO mice revealed that Ziz2 was intrinsically required for MZ B cell development, but not for mature follicular B cells. However, immune responses against NP-CGG (T cell-dependent), TNP-LPS (T cell-independent, TI, type I), and TNP-Ficoll (TI, type II) were not altered in KO mice. We finally demonstrated that CD1d-positive MZ B cell region outside CD169-positive marginal metallophilic macrophages (MMM) was narrowed in Ziz2 KO mice. Furthermore, MMM morphology appeared to be altered in Ziz2 KO mice.

In conclusion, we herein showed that Ziz2 was associated with early bone marrow B cell development, MZ B cell formation, MZ B number/localization around MZ, and MMM morphology which may explain in part the mechanism underlying immuno-senescence.

**Electronic supplementary material:**

The online version of this article (doi:10.1186/s12979-015-0028-x) contains supplementary material, which is available to authorized users.

## Introduction

We originally cloned and identified [1] murine Zizimin2 (Ziz2, Dock11) as a guanine nucleotide exchange factor (GEF) for Cdc42, one of the major Rho GTPases, and demonstrated that it activated the formation of filopodia in HEK293T cells [2]. Since its expression pattern was previously shown to be restricted in immune tissues such as the spleen, thymus, and lymph nodes [1], we expected Ziz2 to be associated with immune responses and/or lymphocyte development. Moreover, we recently discovered that Ziz2 expression levels in immune tissues were reduced with aging in the mouse [3], suggesting that its expression is associated with the mechanisms responsible for immuno-senescence.

B cells (as well as other hematopoietic cells) are generated in bone marrow (BM) from hematopoietic stem cells. BM B cells grow in BM and migrate into the splenic B cell region. In this region, immature B cells grow into two types of mature B cells; mature follicular B cells (“M”), which accumulate inside follicles along with the CXCL13 gradient through CXCR5, and marginal zone (MZ) B cells (“MZ B”), which stall around MZ through the S1PR1/3-S1P and/or integrin signaling pathway [4]. Another B cell population, so called B1a, is also generated in BM, but is located in the peritoneal cavity. Mature follicular B cells are responsible for antibody production in acquired immunity, whereas MZ B and B1a B cells are involved in the protection process against infectious diseases by bridging the time lag between innate and acquired immune response. Aged mice have been shown to have lower numbers of MZ B cells and, thus, are more susceptible to infectious diseases [5]. The mechanisms underlying the age-dependent decline in MZ B cells includes reductions in MZ macrophages and the altered positioning of sinus lining cells and marginal metallophilic macrophages around MZ. Aging also causes defects in BM B cell development [6].

Previous studies on Rho GTPases, such as Cdc42, RhoA, and Rac2, clearly demonstrated that they functioned in B cell development and immune responses by regulating B cell proliferation, survival, and migration, respectively [7-9]. Thus, we expected Ziz2 to also function in these events through its GEF activity. However, the functions of Ziz2 have not yet been fully examined *in vivo*. To gain insights into the mechanisms underlying immuno-senescence, we generated Ziz2 knock out (KO) mice and examined the functions of Ziz2 in B cell development and immune responses. We also obtained Zizimin3 (Ziz3; Dock10) KO mice and examined its functions.

In the present study, we demonstrated that Ziz2 was associated with early BM B cell development, MZ B cell formation and localization around MZ, and thymic CD4^+^ T cell formation, which may explain in part the mechanism responsible for immuno-senescence.

## Results and discussion

### Generation of Ziz2 or Ziz3 KO mice

**Figure 1:**
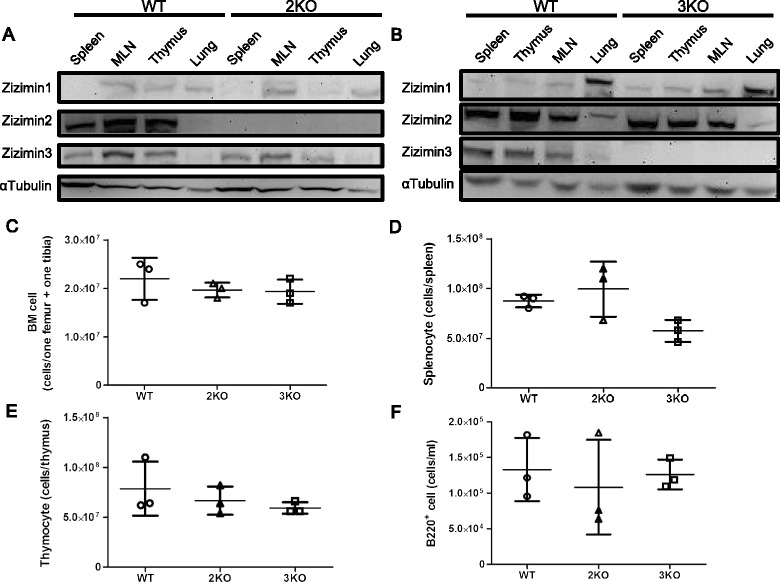
**Absence of Zizimin proteins, but normal numbers of hematopoietic cells in KO mice. (A-B)** Western blotting for Ziz2 **(A)** or Ziz3 **(B)** Protein lysates (30 μg/lane) were loaded and designated antibodies were reacted with the membranes. Replicated membranes were used for all antibodies. Zizimin proteins were absent in KO mice (8 weeks old, male). **(C-F)** The numbers of hematopoietic cells were counted after the hemolytic reaction **(C-E)** or flow cytometric analysis **(F)**. No significant difference was observed among the groups **(C-F)**. Three mice (8 weeks old, female) per group from three independent experiments (one mouse per group per experiment) were used. 2KO: Ziz2 KO. 3KO: Ziz3 KO.

We successfully generated and maintained Ziz2 or Ziz3 KO mouse line. We confirmed the absence of the protein product of either gene in the KO mouse by western blotting (Figure 1A-B). Both KO mice were viable and fertile. In addition, no malformations were evident in KO mice. Thus, these results indicated that Ziz2 or Ziz3 has no critical role in regulating viability, fertility, or malformations in mice.

### Flow cytometric analysis

**Figure 2:**
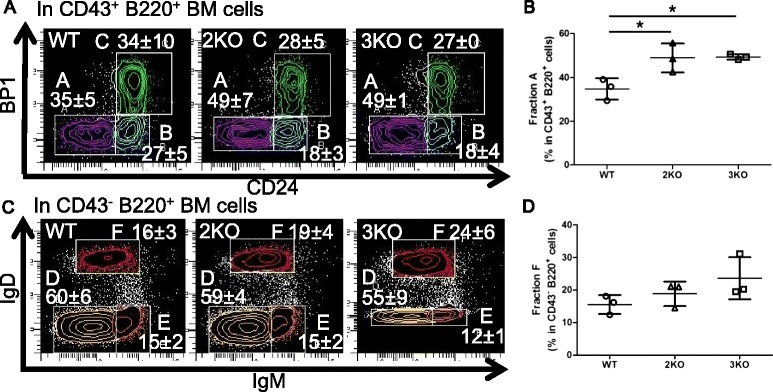
**Early bone marrow B cell development was altered in KO mice. (A)** Cell from BM were analyzed by flow cytometry for fractions “A-C” in CD43^+^ B220^+^ BM cells. Fraction “A” (Germline Pro-B): CD24^−^ BP1^−^. Fraction “B” (DJ-rearranged Pro-B): CD24^+^ BP1^−^. Fraction “C” (Pre-B early): CD24^+^ BP1^+^. **(B)** The percentage of fraction “A” was increased in KO mice. **(C)** Cells from BM were also analyzed by flow cytometry for fractions D-F in CD43^−^ B220^+^ BM cells. Fraction “D” (Pre-B late): IgM^−^ IgD^−^. Fraction “E” (Newly-formed B): IgM^+^ IgD^−^. Fraction “F” (Follicular-type recirculating B): IgM^+^ IgD^+^. **(D)** The percentage of fraction “F” was not altered in KO mice. 2KO: Ziz2 KO. 3KO: Ziz3 KO. *: P < 0.05 Three mice (8 weeks old, female) per group from three independent experiments (one mouse per group per experiment) were used.

**Figure 3:**
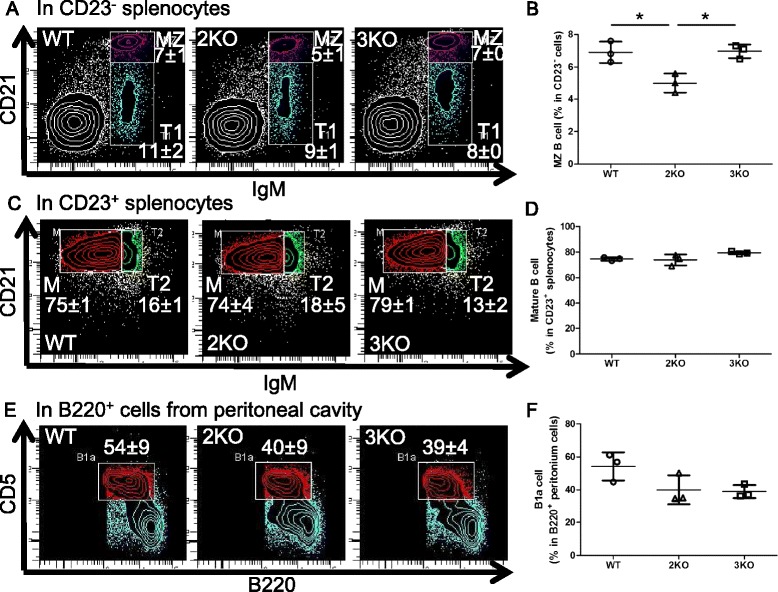
**The percentage of marginal zone B cells was decreased in Ziz2 KO mice. (A)** Splenocytes were analyzed by flow cytometry for T1 and MZ B cells in CD23^−^ cells. T1: IgM^+^ CD21^−^. MZ: IgM^+^ CD21^+^. **(B)** The percentage of MZ B cells was decreased in Ziz2 KO mice. **(C)** Splenocytes were analyzed by flow cytometry for T2 (Transition 2) and mature (M) B cells in CD23^+^ cells. T2: IgM^high^ CD21^+^. M: IgM^low/middle^ CD21^+^. **(D)** The percentage of “M” was not altered in KO mice. **(E)** Cells from the peritoneal cavity were analyzed by flow cytometry for B1a B cells in B220^+^ cells. B1a: B220^middle^ CD5^middle^. **(F)** The percentage of B1a cells was not altered among the groups. 2KO: Ziz2 KO. 3KO: Ziz3 KO. *: P < 0.05 Three mice (8 weeks old, female) per group from three independent experiments (one mouse per group per experiment) were used.

**Figure 4:**
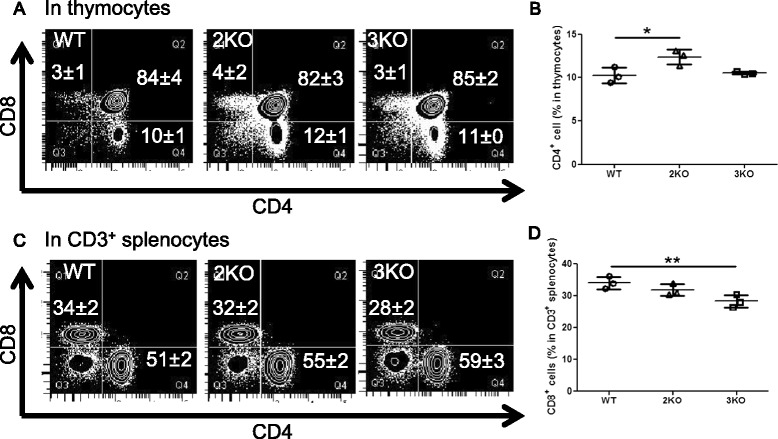
**Thymic or splenic T cells were changed in Ziz2 or Ziz3 KO mice. (A)** Thymocytes were analyzed by flow cytometry for double negative (CD4^−^ CD8^−^), double positive (CD4^+^ CD8^+^), CD4 single positive (CD4^+^ CD8^−^), or CD8 single positive (CD4^−^ CD8^+^) T cells. **(B)** The percentage of CD4(+) cells in thymocytes was increased in Ziz2 KO mice. **(C)** Splenocytes were analyzed by flow cytometry for CD4^+^ or CD8^+^ T cells in CD3^+^ cells. **(D)** The percentage of CD8^+^ cells in CD3^+^ splenocytes was decreased in Ziz3 KO mice. 2KO: Ziz2 KO. 3KO: Ziz3 KO. *: P < 0.05 **: P < 0.01 Three mice (8 weeks old, female) per group from three independent experiments (one mouse per group per experiment) were used.

We next analyzed B and T cell development in KO mice. We initially counted cells from BM, the spleen, thymus, or peritoneal cavity, and then stained them for the flow cytometric analysis. We noted that the numbers of viable (Trypan blue negative) cells were not affected by the deletion of the Ziz2 or Ziz3 gene (Figure 1C-F). In the flow cytometric analysis, each fraction in the developmental course of B or T cells in BM, the spleen, thymus, or peritoneal cavity was examined. We assessed fractions “A” to “F” for BM B cells [10], as shown in Figure 2A and C. B220^mid^ CD5^mid^ cells [11] were checked for B1a cells from the peritoneal cavity (Figure 3E). T1 (Transition 1), T2, M (mature follicular), and MZ B cell fractions [12] were analyzed for splenic B cells (Figure 3A and C). CD4^+^ and CD8^+^ fractions were analyzed in total thymocytes for thymic T cells (Figure 4A). Regarding splenic T cells, CD4^+^ and CD8^+^ cell fractions were examined in CD3^+^ splenocytes (Figure 4C). The results showed that the percentage of fraction “A” in BM B cells was higher in Ziz2 or Ziz3 KO mice than in wild type (WT) mice (Figure 2A-B). This may be explained by the reduction of immature B cell development as shown in the KO mice for Cdc42, which is a target of Ziz2 and possibly of Ziz3, because hematopoietic stem cell (HSC)-specific Cdc42 KO was previously shown to result in a reduction of Pro/Pre/immature B cells [7]. On the other hand, other fractions in BM B cells were not affected in KO mice (Figure 2C-D), which was contrary to the phenotype of Cdc42 KO mice [7]. These results indicated that Ziz2 or Ziz3 assisted in BM B cell development from fractions “A” to “B” possibly through Cdc42. These results also suggested that Cdc42 activation required not only Ziz2 or Ziz3, but also other GEFs for their role in BM B cell proliferation because HSC-specific Cdc42 KO was found to result in significant reductions in all other fractions of BM B cells.

**Figure 5:**
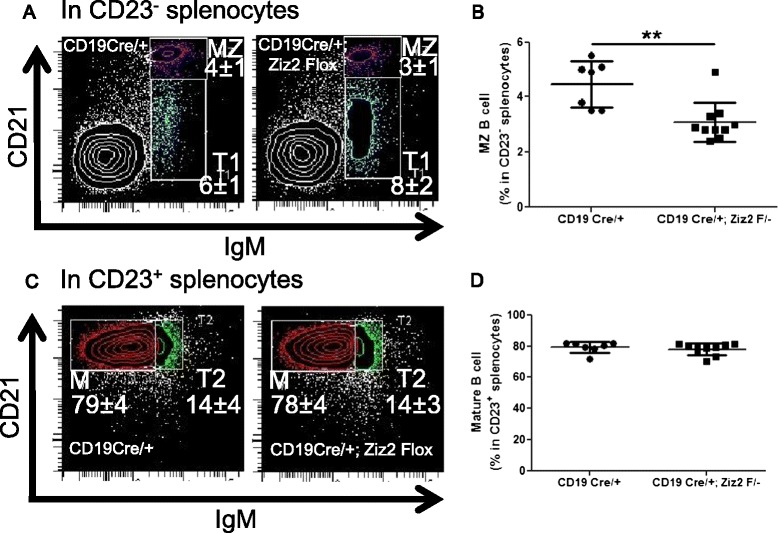
**Reduction in marginal zone B cell percentage in B cell-specific Ziz2 KO mice. (A)** Splenocytes were analyzed by flow cytometry for T1 (Transition 1) and MZ B cells in CD23^−^ cells. T1: IgM^+^ CD21^−^. MZ: IgM^+^ CD21^+^. **(B)** The percentage of MZ B cells was lower in CD19^Cre/+^; Ziz2^flox^ (Conditional KO) mice than in CD19^Cre/+^ (Control) mice. **(C)** Splenocytes were analyzed by flow cytometry for T2 and mature (M) B cells in CD23^+^ cells. T2: IgM^high^ CD21^+^. M: IgM^low/middle^ CD21^+^. **(D)** The percentage of “M” was not altered in KO mice. **: P < 0.01 Between seven and ten mice (9–11 weeks old, male or female) per group from five independent experiments (from one to three mice per group per experiment) were used.

Although the percentage of B1a cells was not altered in KO mice (Figure 3E-F), the percentage of MZ B cells was reduced in Ziz2 KO mice, but not in Ziz3 KO (Figure 3A-B). The percentage of fraction “M” in splenic B cells was not affected in KO mice (Figure 3C-D). In addition, B cell-specific Ziz2 KO resulted in a reduction in the MZ B cell percentage, but not that of mature follicular B cells (Figure 5A-D). These results indicated that MZ B cells, but not mature follicular B cells, intrinsically required Ziz2 for proper development.

Regarding T cell development, the percentage of thymic CD4^+^ T cells was increased in Ziz2 KO mice (Figure 4A-B). On the other hand, the percentage of splenic CD8^+^ T cells was decreased in Ziz3 KO mice (Figure 4C-D). These results suggested that Ziz2 suppressed thymic CD4^+^ T cell formation, whereas Ziz3 assisted in splenic CD8^+^ T cell formation. Interestingly, histological examination revealed that global architectural features in thymus and spleen seemed to be indistinguishable between WT and the KO mice (Additional file 1: Figure S1). However, T cell development in thymus or spleen may be altered in Ziz2 or Ziz3 KO mice, respectively (Figure 4A-D). Thus, Ziz2 or Ziz3 may affect T cell development, at least, in the cellular level. For example, T cells are matured in thymus while moving from sub-capsular region to medulla through contacting with other cortical epithelial cells, dendritic cells, or macrophages. In this condition, Ziz2 may function upon contacting T cells with other cells and as described above to form CD4^+^ T cells in thymus through generating filopodiae. On the other hand, Ziz3 may function upon invade from thymus into blood stream or from blood stream into spleen because T cell development in Ziz3 KO thymus was intact and a literature has demonstrated its function in amoeboid invasion in melanoma cells [13].

Taken together, the results of the flow cytometric analysis revealed that Ziz2 was associated with BM Pro B cell, MZ B cell, and thymic CD4^+^ T cell formation. Ziz3 may also be associated with BM Pro B cell and splenic CD8^+^ T cell formation. GTPases such as Cdc42 and RhoA are known to be involved in B cell development; however, it has not yet been established whether GTPases are involved in T cell development in mice. We here demonstrated that T cell development was altered in Ziz2 or Ziz3 KO mice. Thus, Ziz2 or Ziz3 may regulate T cell development in the thymus or spleen through Cdc42 or other GTPases.

### Immunization with T cell-dependent or T cell-independent antigens

**Figure 6:**
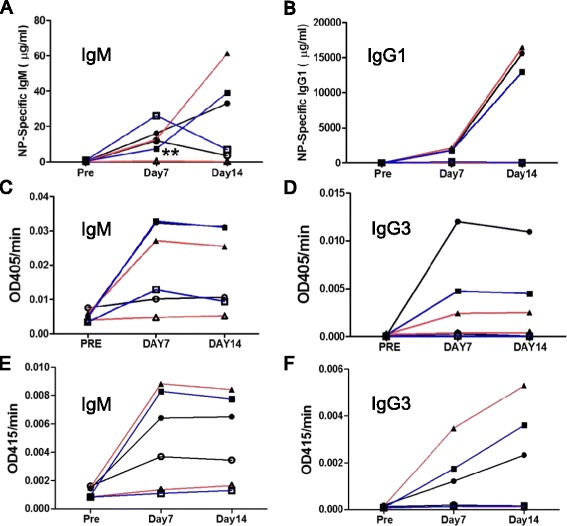
**Immune responses were not altered in KO mice. (A-B)** Antigen(NP-CGG)-specific antibody titers [IgM in **(A)**, IgG1 in **(B)**] in WT and KO mice. Eight mice per group were used for the antigen-injected groups. **(C-D)** Antigen(TNP-LPS)-specific antibody titers [IgM in **(C)**, IgG3 in **(D)**]. Between eight and eleven mice per group were used for the antigen-injected groups. **(E-F)** Antigen(TNP-Ficoll)-specific antibody titers [IgM in **(E)**, IgG3 in **(F)**]. Between six and eight mice per group were used for the antigen-injected groups. Circles: WT, Triangles: Ziz2KO, Squares: Ziz3 KO, Open symbols: PBS-injected groups, Closed symbols: antigen-injected groups. **: P < 0.01.

Since the percentage of B or T cells in the spleen or thymus was altered in KO mice, we next examined immune responses in these mice. NP-CGG was used for the T cell-dependent (TD) antigen stimulation, and NP-specific antibody concentrations in serum were measured by ELISA. TNP-LPS (as type I) or TNP-Ficoll (as type II) was used for the T cell-independent (TI) antigen stimulation, and TNP-specific antibody titers (OD415/min) were measured by ELISA. We expected altered immune responses in KO mice, especially for TI antigens, because the number of MZ B cells, which are associated with the production of antibodies against TI antigens, was reduced in KO mice. However, no significant difference was observed in antibody titers between WT and KO mice (Figure 6A-F; except for WT vs. Ziz3KO on day 7 for NP-CGG, as shown in Figure 6A). Although the immune response (IgM antibody titer) of Ziz3 KO for NP-CGG was weaker than that of WT on day 7, the response of Ziz3 KO mice against PBS appeared to be similar. Thus, we assumed that the significant difference observed on day 7 may have been caused by a technical deviation. Taken together, these results indicated that Ziz2 or Ziz3 was not associated with antibody production against TD and TI antigens. GTPase KO mice, such as Cdc42 or Rac2, have been reported to have reduced B cell numbers and weaker immune responses [7,9]. Thus, these findings also indicated that Ziz2 or Ziz3 may not be associated with Cdc42 or Rac2 for TD or TI immune responses.

### Histological examination of MZ B cells

**Figure 7:**
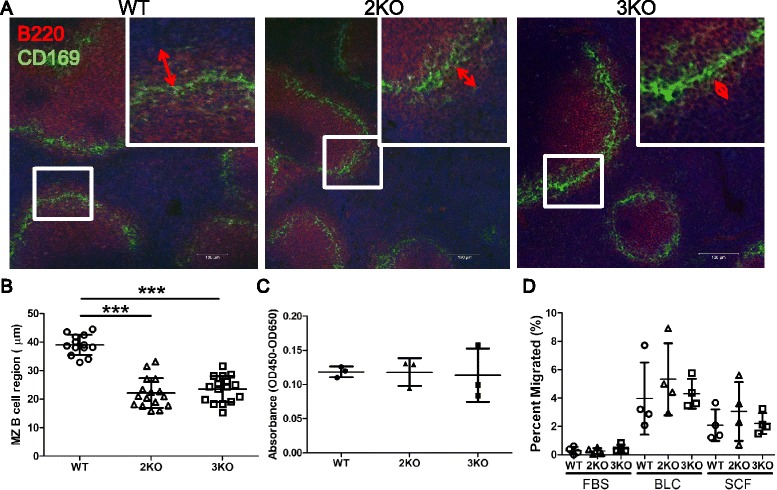
**Marginal zone B cell regions were narrowed in Ziz2 KO mice. (A)** Spleen sections were stained with anti-B220 (Red) and anti-CD169 (Green) antibodies for the MZ B cell region (B220-positive region outside CD169-positive cells). All mice were 10 weeks old. Scale bars: 100 μm. **(B)** MZ B cell regions were narrower in Ziz2 and Ziz3 KO mice than in wild type mice. **(C)** The proliferative activity of MZ B cells in response to LPS was not altered in both KO mice. Three mice per group were used. Data from three independent experiments (one mouse per group per experiment was used) were summarized. **(D)** The migratory activity of MZ B cells was analyzed using a transwell and flow cytometry. Activity against BLC or SDF1 (SDF) was not altered in both KO mice. Four mice per group were used. Each plot indicates data from one mouse. Data from four independent experiments (one mouse per group per experiment was used) were summarized. 2KO: Ziz2 KO. 3KO: Ziz3 KO. ***: P < 0.0001.

**Figure 8:**
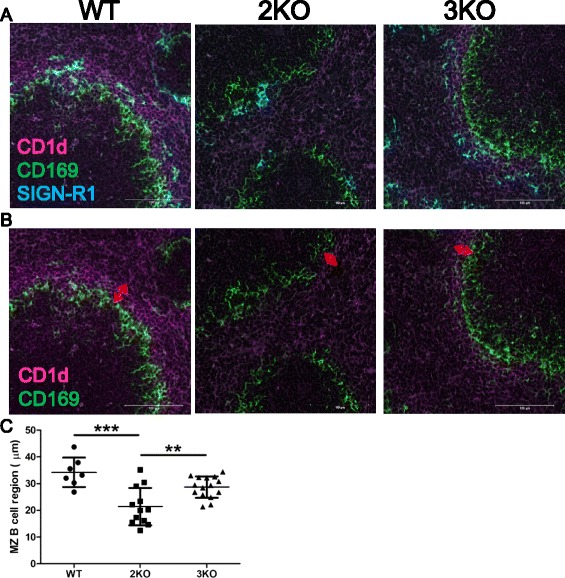
**Marginal zone B cell regions were narrowed in Ziz2 KO mice. (A-B)** Splenic sections were stained with anti-CD1d (Magenta), anti-CD169 (Green) **(B)**, and anti-SIGN-R1(Blue) antibodies **(A)** Three mice per group were used. Between one and eight follicles per mouse (per section) were captured. All mice were 10 weeks old. Scale bars: 100 μm. **(C)** The CD1d-positive region outside CD169-positive cells was significantly narrower in Ziz2 KO mice than in the other groups. 2KO: Ziz2 KO. 3KO: Ziz3 KO. **: P < 0.01 ***: P < 0.0001 N = 7–15.

To examine histological features in the Ziz2 or Ziz3 KO spleen, especially for MZ B cell development, frozen sections were stained with anti-B220 and anti-CD169 antibodies for B cells and marginal metallophilic macrophages (MMM), respectively. The MZ B cell region (B220^+^ region outside of CD169^+^ cells) was significantly narrower in KO mice than in WT mice (Figure 7A-B). The reduction in the MZ B cell region in Ziz2 KO mice was also confirmed by staining with another antibody specific for CD1d, a marker of MZ B cells (Figure 8A-C). These results indicated that Ziz2 or Ziz3 was associated with MZ B cell localization around splenic MZ. S1PR1, S1PR3, LFA1, VLA4, ICAM1, VCAM1, PYK2, LSC, DOCK2, RAC2, and MARCO are known to be involved in MZ B cell localization [4]. After being stimulated by surrounding S1P (sphingosine-1-phosphate; a ligand for S1PR1 and S1PR3) in blood or by MARCO expressed on the cell surface of MZ macrophages (MZM), MZ B cells stall in MZ due to an interfering powerful attraction towards follicles through CXCR5-CXCL13 signaling. In addition, integrin signaling-associated gene (e.g. Pyk2, Lsc, Dock2, or Rac2) KO mice have a normal number of mature follicular B cells, but a low numbers of MZ B cells and poor antibody responses to antigens [4,9,14-16]. In these conditions, our results may suggest that Ziz2 functions in MZ B cell retention around MZ through protruding filopodiae and utilizing integrin molecules by activation of S1P/S1PR and/or MARCO signaling pathway. In the present study, we also examined histologically SIGN-R1^+^ MZMs and CD169^+^ MMMs that are important surrounding partners for MZ B cells, for example, in TI antigen presentation. The staining pattern indicated that Ziz2 may be involved in forming dense MMM ring around MZ because MMM ring around MZ was sparse in Ziz2 KO mice (Figure 8A and Additional file 2: Figure S2A). On the other hand, not only MMM number but also MZM number seemed to be indistinguishable between WT and Ziz2 KO mice (Additional file 2: Figure S2B). MZM number is known to correlate with MZ B cell number [5]. In this study, however, MZ B cell number is reduced in Ziz2 KO mice with no alteration in MZM number. We reasoned that Ziz2 possibly has an important role to generate and localize MZ B cells around MZ through connecting MZ B cells and MZMs by filopodiae [4]. Taken together, our results indicated that Ziz2 was crucially involved in MZ B cell formation by regulating MZ B cell localization around MZ and MMM morphology, possibly through filopodiae by activation of S1P/S1PR, integrin, and/or MARCO signaling pathway from MZM.

### Proliferation assay

Since the percentage and localization of MZ B cells were decreased in KO mice, we next analyzed the proliferative activity of MZ B cells *in vitro*. In the proliferation assay, flow cytometry-sorted MZ B cells were incubated with LPS and assessed by a colorimetric method (Figure 7C). Contrary to our expectation, the results showed that proliferative activity was not significantly different between WT and KO mice. These results suggested that neither Ziz2 nor Ziz3 had any obvious function in the proliferation of MZ B cells upon LPS stimulation. Moreover, Ziz2 or Ziz3 may not be associated with Cdc42 at least in B cell proliferation, as Cdc42 regulates B cell proliferation.

### Migration assay

We examined the migratory activity of splenic B cells from KO mice *in vitro* towards BLC or SDF1 in order to address the reduction in the number of MZ B cells in KO mice. To assess migratory activity, all splenocytes were loaded into the upper chamber of a transwell and BLC or SDF1 was added to the lower chamber. Percent migration was analyzed by counting the cell number of each fraction of B cells in the input and lower chamber by flow cytometry. However, no significant difference was observed in the migratory activities of WT and KO (Figure 7D). Taken together, these data indicated that Ziz2 was not associated with MZ B cell migration towards BLC and SDF1. In other words, reduction of MZ B cells in Ziz2 KO mice may be caused, at least, by alteration of MZ B cell localization around MZ and/or MMM morphology.

## Conclusion

Regarding an association between MZ B cells and susceptibility against infectious diseases especially in aging process, previous paper demonstrates that MZ B cell/MZM number and localization of MMM/sinus lining cells around MZ are changed upon aging in mice and it can be one of the cause of age-associated higher susceptibility against infectious diseases [5,17]. MMM may also activate CD1d-restricted invariant natural killer T cells to promote rapid antibody response via extra-follicular B cells [4]. In this study, MZ B cell reduction (Figure 3A) and sparse MMM (Figures 7A and 8B, and Additional file 2: Figure S2A) were observed, but not MZM reduction (Additional file 2: Figure S2B), in Ziz2 KO mice. In addition, we observed that Ziz2 expression level is decreased along with aging in splenic B cells (reducing tendency was also observed in DC and T cells, but not NK cells) (Additional file 3: Figure S3). Thus, it is warranted in the future to test if the expression level of Ziz2 in MZ B cells / MMM reduces with aging, possibly causing MZ B cell number decline and morphological change of MMM. Nevertheless, Ziz2 KO mice did not show any significant difference in relatively early phase (from day 7 to 14) of immune response against TD or TI antigens as compared to WT mice (Figure 6). From this point of view, we could not conclude that Ziz2 is associated with the immune responses (also with the susceptibility against infectious diseases). However, because MZ is also important for long-lived memory B cell accommodation for T-cell dependent antigens [17] which is generated in relatively late phase (from day 28 to 35) of the immune response , we are now focusing on the Ziz2 function in memory B cell formation.

Regarding functional similarity between Ziz2 and Ziz3, we initially expected that Ziz2 and Ziz3 have the same function *in vivo* because of its structurally similarity. Although we observed functional similarity in BM B cell development, we also observed functional differences in MZ B cell formation/localization, thymic CD4^+^ T cell formation, and splenic CD8^+^ T cell formation. It is possible that Ziz2 or Ziz3 is expressed in different type of cells in BM but same phenotype was observed in both KO mice. It is also possible that upstream regulatory factor(s) may be different for Ziz2 and Ziz3, because IL4 up-regulates Ziz3 but not Ziz2 in human B cells [18]. For these issues, we are now trying to identify the upstream transcriptional factor(s) by using reporter assay with their putative promoter regions.

Taken together, we herein demonstrates that Ziz2 is associated with early BM B cell development (from Fractions “A” to “B”), MZ B cell formation and localization around MZ, thymic CD4^+^ T cell formation. On the other hand, Ziz3 was associated with early BM B cell development (from Fraction “A” to “B”) and splenic CD8^+^ T cell formation. These results also indicated that the age-associated decline in Ziz2 may affect MZ B cell formation/localization around MZ and MMM morphology that will potentially affect susceptibility for infectious diseases.

## Methods

### Generation of Ziz2 or Ziz3 KO mice

We generated Ziz2 KO mice using the Cre-loxP system. Briefly, we inserted a targeting vector that had the Ziz2 exon1 sequence flanking two loxP sequences into murine ES cells, transferred it into blastocysts, then transplanted the blastocysts into the uterus of a pseudo-pregnant foster mother. Chimera mice were mated with WT mice to obtain flox mice. To obtain conventional KO mice, flox mice were mated with CAG-Cre mice. Concerning Ziz3, we obtained frozen embryo from the European Mouse Mutant Archive (EMMA) and transferred to generate Ziz3 KO mice. To generate B cell-specific conditional KO mice, floxed Ziz2 mice were mated with CD19 Cre/+ knock in mice. To confirm genotypes by PCR, the following primers were used; murine Ziz2 KO mice typing 5′-CGA TGT GTA TCC GCA CTC AA-3′ (sense) and 5′-CAC AGC GGG TTT CAC AGA G-3′ (antisense for floxed and WT allele) or 5′-GTC CAT TCC CAT GCT CTT GT-3′ (antisense for deleted allele); murine Ziz3 KO mice typing 5′-TCA ACC CAT TTC TGA CCT CC-3′ (sense) and 5′- GTT CTG TGG CAC TGG AGG AT-3′ (antisense for floxed and WT allele) or 5′-AGC GCA TCG CCT TCT ATC GC-3′ (antisense for deleted allele); CD19Cre 5′-AGA AGT CCT TAC TGG TGG AGG TAG-3′ (sense) and 5′-AGC CCG GAC CGA CGA TGA AG-3′ (antisense). In this study, 8–12 weeks old mice were used.

### Western blotting

Western blotting was performed as described previously [19]. Briefly, a protein lysate was prepared by homogenizing tissues with the IKA T10 basic hand mixer in RIPA buffer (25 mM Tris–HCl pH7.6, 150 mM NaCl, 1 mM EDTA, 1% NP-40, 1% Na-deoxycholate, 0.1% SDS, with protease inhibitor; Roche, 04 693 132 001) and centrifuging (13,000 g, 4°C, 20 minutes). Protein concentrations were determined by the BCA assay (Thermo, 23227). Thirty micrograms of the protein lysate was loaded onto a 3-10% gradient gel (196–14621, Wako, Osaka, Japan) and separated by electrophoresis at 20 mA (constant current) per gel for 60 minutes. After semi-dry blotting at 2 mA per cm^2^ for 80 minutes into Hybond-P (GE, Amersham, RPN303F), the membrane was incubated with 1% skimmed milk/PBST (0.1% Tween20/PBS) for blocking while shaking at approximately 60–70 rpm in a cold room overnight (around 16 hours). The first antibody [For Zizimin1 (Dock9): Rabbit polyclonal, NB500-265, Novus, 1:500; For Ziz2: Rat monoclonal, 214I9 [20], 1:100; For Ziz3: Rabbit polyclonal, NB100-60669, Novus, 1:2000; For αTubulin: Mouse monoclonal, T6074-200UL, Sigma, 1:2500] was diluted with the blocking buffer and incubated with the membrane for 2 hours at room temperature while shaking horizontally at 60–70 rpm. The appropriate secondary antibodies conjugated with HRP (horseradish peroxidase) were incubated with the membrane, as described for the first antibody. Signals were detected by a chemiluminescent method (Immobilon, WBKLS0500, Millipore).

### Flow cytometric analysis

A flow cytometric analysis was performed as described elsewhere [19]. Briefly, cells from bone marrow (BM), the spleen, thymus, or peritoneal cavity were hemolyzed with hemolytic buffer (150 mM NH_4_Cl, 10 mM KHCO_3_, 0.1 mM EDTA, pH 7.3) and the viable cell number was counted using the Trypan blue (0.1% by PBS) exclusion method on a hemocytometer. The hemolyzed cells were incubated with anti-CD16/32 (BioLegend, 101302, 1:100) for blocking on ice for 5 minutes. The primary antibodies were then incubated with the cells on ice for 10 minutes. The primary antibodies were as follows; anti-CD3-biotin (BD, 553060, 1:100), anti-CD4-APC (allophycocyanin) (BioLegend, 100515, 1:8000), anti-CD5-APC (BD, 550035, 1:200), anti-CD8-FITC (fluorescein isothiocyanate) (BioLegend, 100705, 1:5000), anti-CD21-PE (phycoerythrin) (BioLegend, 123409, 1:80), anti-CD23-biotin (BioLegend, 101603, 1:200), anti-CD24-biotin (eBioscience, 13-0242-81, 1:300), anti-CD43-FITC (eBioscience, 11-0431-81, 1:1000), anti-B220-APC (BioLegend, 103211, 1:160), anti-B220-PE (BioLegend, 103207, 1:80), anti-BP1(Ly51)-PE (BioLegend, 108307, 1:80), anti-IgD-PE (BioLegend, 405705, 1:500), anti-IgM-PE Cy7 (BioLegend, 406513, 1:400). Before being analyzed or sorted, the stained cells were re-suspended in 7AAD (BioLegend, 420404, 1:60) diluted in 1% BSA/PBS, and 7AAD-positive dead cells were excluded from the experiments. The appropriate secondary reagents were applied [Streptavidin-APC (eBioscience, 17-4317-82, 1:1000), Streptavidin-PE Cy7 (BD, 557598, 1:1000 for BM cells or 1:9000 for T cells)]. An analysis or sorting was performed with FACS AriaII (BD) or FACS Cnato II (BD), respectively. The absolute numbers of cell populations from the peritoneal cavity were divided by the volume (ml) of the medium (10% FBS/RPMI 1640), which was collected from the peritoneal cavity.

### Histological examination

A histological examination was performed as described recently [19]. In addition, anti B220-eFluor570 (eBioscience, 41-0452-80, 1:200) or CD1d-Pacific Blue (BioLegend, 123516, 1:50) was applied to the frozen sections with CD169-FITC (AbD Serotec, MCA947F, 1:100) and SIGNR1-APC (eBioscience, 17-2093-80, 1:20). Image J (NIH, USA) was used to measure the MZ B cell region. One marginal zone B cell region was chosen per figure. The maximum width (along with the radial axis from around the center point of white pulp) of the MZ B region per figure was selected for data sampling. Between 3 and 4 mice per group were analyzed.

### Immunization and serum preparation

Regarding NP-CGG, 100 μg (per 100 μl of PBS per mouse) of NP_30_-CGG (4-Hydroxy-3-nitrophenylacetyl conjugated with chicken gamma globulin, Biosearch Technologies, N-5055D-5) was incubated with aluminum hydroxide/magnesium hydroxide (100 μl per mouse, Imject Alum Adjuvant, Thermo, 77161) for 60 minutes at approximately 20 rpm with a rotator at room temperature, then 200 μl (100 μg of NP-CGG) per mouse of the antigen solution was administered. Ten micrograms (per 200 μl of PBS per mouse) of TNP_40_-Ficoll (2,4,6-Trinitrophenyl conjugated with amino-ethyl-carboxy-methyl ficoll, Biosearch Technologies, F-1300-10) was used for TNP-Ficoll. Twenty micrograms (per 200 μl of PBS per mouse) of TNP_0.5_-LPS (2,4,6, Trinitrophenyl conjugated with lipopolysaccharide, Biosearch Technologies, T-5065-1) was used for TNP-LPS. All antigens (200 μl per mouse) were injected into the peritoneal cavity.

Blood was collected from the tail vein at the designated time points. The collected blood in a 1.5 ml tube was settled in a refrigerator overnight then centrifuged at 1,000 g for 20 minutes at 4°C. The supernatant was used as serum.

### Enzyme-linked immunosorbent assay

An enzyme-linked immunosorbent assay (ELISA) was performed as described previously [19]. NP-BSA (Biosearch Technologies, N-5050 M-10, 10 μg/ml, 45 μl per well) was used as an antigen for the NP-specific antibody. To assess the antibody titer, the velocity of optical density (OD415/min, for TNP-specific antibody) or concentration (μg/ml, for NP-specific antibody) was calculated. To calculate the NP-specific antibody titer, a standard curve was made with anti-NP antibodies (N1G9 for IgG1 [21] or 267.7 μ for IgM [22]).

### Proliferation assay

To assess the proliferative activity of MZ B cells from WT or KO mice, MZ B cells (CD23^−^ CD21^high^ IgM^+^ splenocytes) were sorted by flow cytometry and incubated (10^5^ cells/well of a 96-well plate) for 3 days with 20 μg/ml of LPS (Sigma, L2630-25MG) diluted in 10% FBS/RPMI1640. Proliferative activity was assessed by a colorimetric assay (Wako, Dojindo, Cell Counting Kit 8, 347–07621). The incubation time was 6 hours at 37°C, 5% CO_2_. OD450 nm was used to detect signals. OD650 nm was used as a background signal.

### Migration assay

The migration assay for MZ B cells was performed as described previously [19], except for slight modifications in the incubation time (4 hours) and recording conditions [with FACS CantoII (BD), flow rate: High for 60 seconds].

### Statistical analysis

Data are presented as the mean ± standard deviation, except for Figure 6, in which only average values are shown to improve the appearance of the data. Differences in the average values among groups were mainly analyzed by a one-way ANOVA with Bonferroni’s multiple comparison test, except for Figure 6B-E, in which a two-way ANOVA with Bonferroni’s multiple comparison test was applied because an interaction between group and time was not detected. P values less than 0.05 was considered significant. These analyses were performed on Prism 5.0 (Version 5.02, GraphPad Software, La Jolla, CA, USA).

## Additional files

Additional file 1: Figure S1. Hematoxylin and eosin (HE) staining of spleen and thymus; Spleen or thymus from 8 weeks old male (WT/2KO) or 14 weeks old female (3KO) mouse was excised and fixed in 10% formalin, overnight in refrigerator. The tissues were embedded in paraffin and sectioned into 2 μm then stained by HE. Representative figures from one (3KO) to two (WT/2KO) mice were shown. Scale bars: 100 μm. 2KO: Ziz2 KO, 3KO: Ziz3 KO. Additional file 2: Figure S2. Marginal metallophilic macrophage (MMM) and marginal zone macrophage (MZM) in Ziz2 KO mice **(A)** Splenic sections from 9–10 weeks old female mice were stained with anti CD169 (Green) antibody. From three to four sections per mouse (one WT mouse and two KO mice were used) were stained and captured multiple area for localization of CD169^+^ cells. Dense CD169^+^ cells that are observed around MZ in WT sample (indicated by red line) seemed sparse in the KO sample (indicated by red-separated lines). Scale bars: 10 μm **(B)** FACS analysis for MZM and MMM. MZM or MMM were analyzed by staining splenocytes with anti SIGNR1 or anti CD169 antibody, respectively. There is no significant difference between the groups. WT: wild type. 2KO: Ziz2 KO. N = 9–18. Additional file 3: Figure S3. Ziz2 expression levels in splenic B/DC/NK/T cells from young (7–9 weeks old) or aged (2 years old) female mice. Splenic B, DC (dendritic cell), NK (natural killer), or T cells were sorted as B220^+^, CD11c^+^, NK1.1^+^, or CD3^+^ cells, respectively. Sorted cells were washed by PBS then immersed by RNA later (Ambion). RNA was extracted from the cells (that were pooled for three mice per group) by using QIAshredder and RNeasy Micro Kit (Qiagen). Reverse transcription was performed by using ReverTra Ace qPCR RT Master Mix with gDNA Remover (Toyobo). Five nano gram per well of cDNA was mixed with Thunderbird qPCR Mix (Toyobo) and TaqMan primer and prove (Assay ID: Mm01297557_m1 for Zizimin2 and Mm99999915_g1 for GAPDH as internal control, Applied Biosystems) then analyzed by PikoReal 96 Real-Time PCR System (Thermo scientific). Relative expression level of Zizimin2 to GAPDH are shown (triplicate of one representative experiment). The expression level of Zizimin2 in B cell was reduced in aged mice. *: P < 0.05 ***: P < 0.0001 N = 3 (triplicate).

## Abbreviations

BM: Bone marrow
GEF: Guanine nucleotide exchange factor
HSC: Hematopoietic stem cell
KO: Knock out
MMM: Marginal metallophilic macrophage
MZ: Marginal zone
MZM: Marginal zone macrophage
TD: T cell-dependent
TI: T cell-independent
Ziz2: Zizimin2
Ziz3: Zizimin3
WT: Wild type
