# Supplementary figures and images for: The immunosenescence-related gene Zizimin2 is associated with early bone marrow B cell development and marginal zone B cell formation
Source: Immun Ageing. 2015 Feb 22;12:1. doi: 10.1186/s12979-015-0028-x (PMC4343071; doi:10.1186/s12979-015-0028-x)

## Slide 1
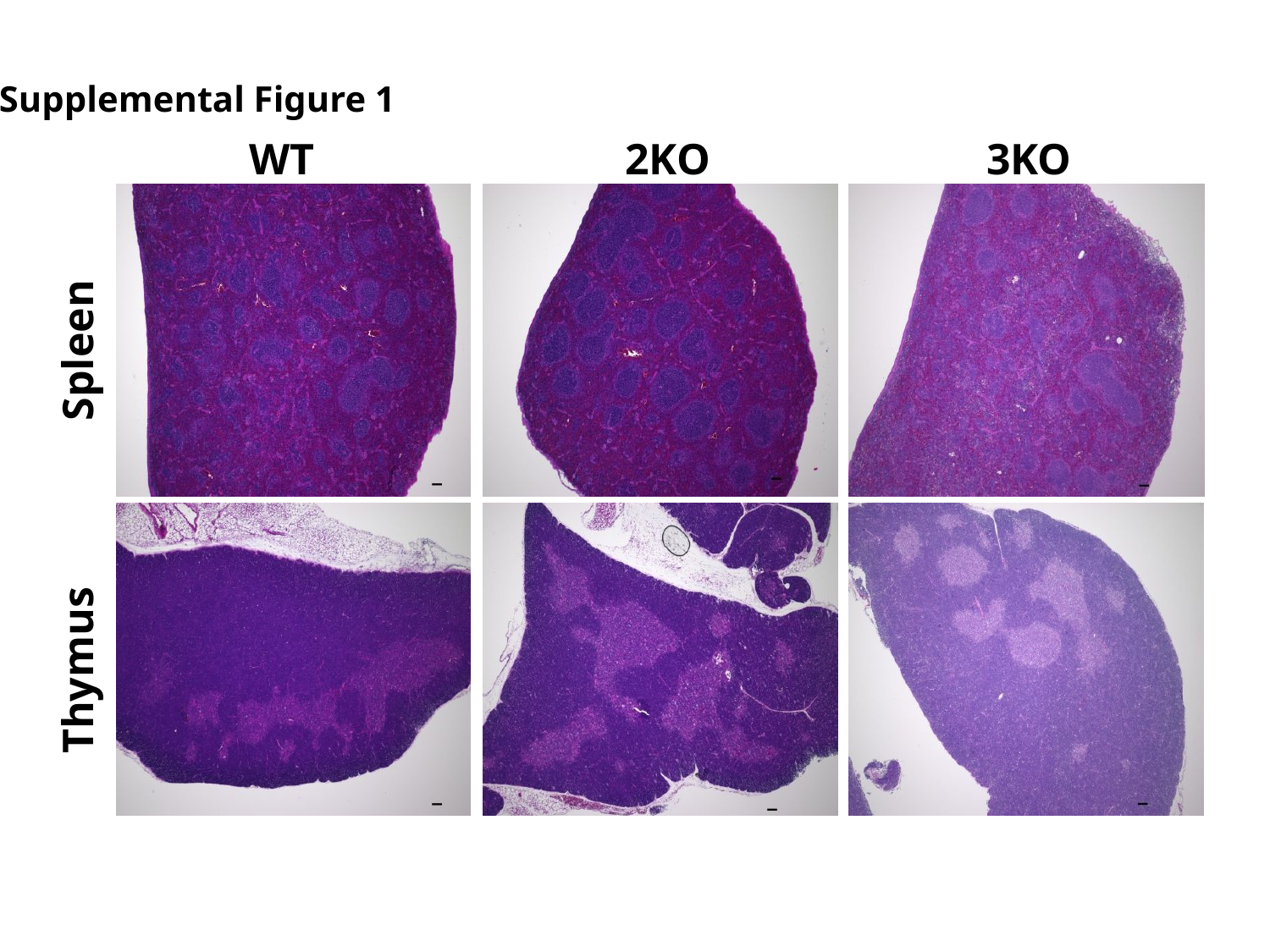

Supplemental Figure 1
WT
2KO
3KO
Spleen
Thymus

Supplement: Additional file 1: Figure S1. — Hematoxylin and eosin (HE) staining of spleen and thymus; Spleen or thymus from 8 weeks old male (WT/2KO) or 14 weeks old female (3KO) mouse was excised and fixed in 10% formalin, overnight in refrigerator. The tissues were embedded in paraffin and sectioned into 2 μm then stained by HE. Representative figures from one (3KO) to two (WT/2KO) mice were shown. Scale bars: 100 μm. 2KO: Ziz2 KO, 3KO: Ziz3 KO. [file 12979_2015_28_MOESM1_ESM.pptx]

## Slide 1
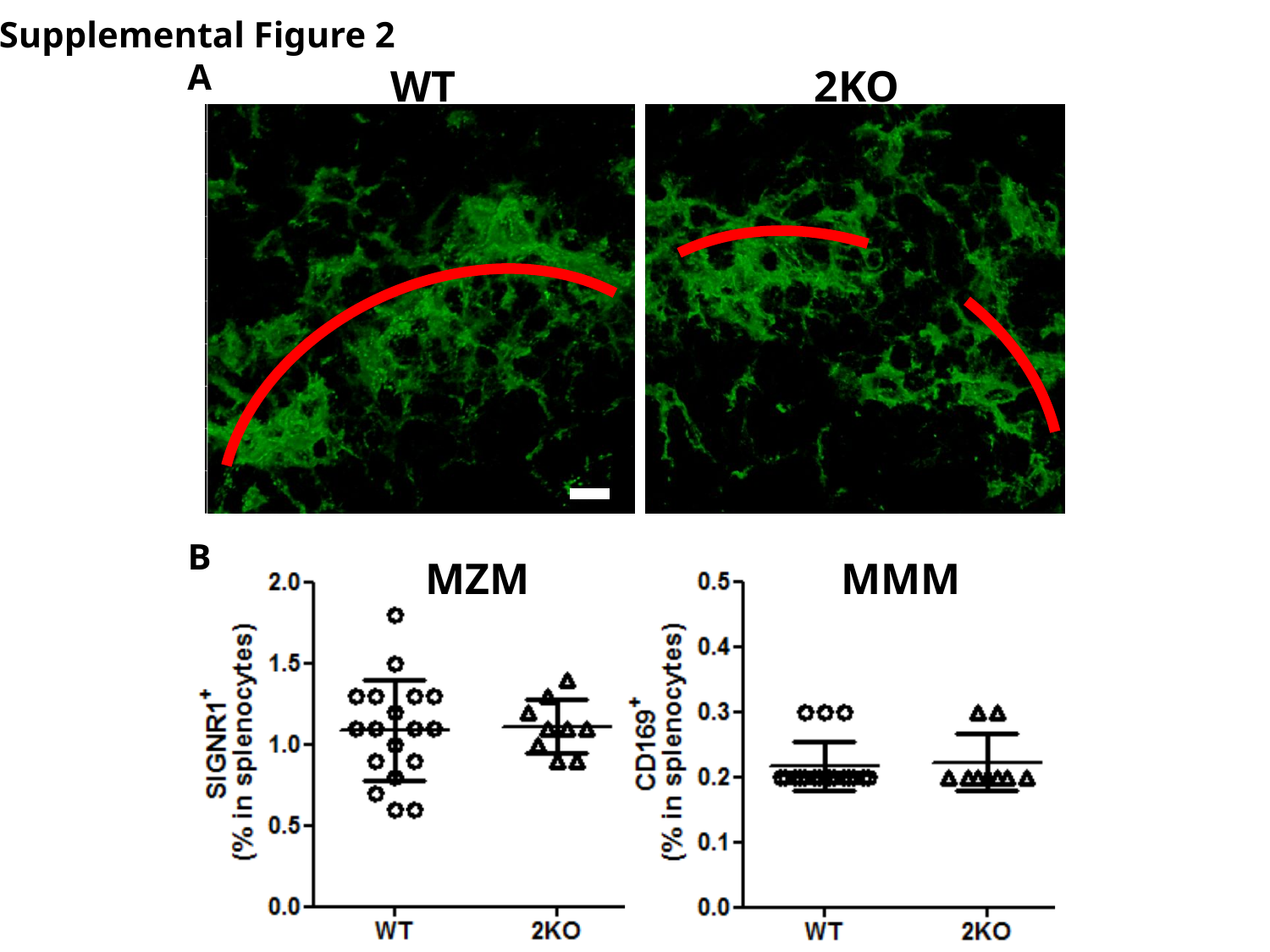

Supplemental Figure 2
A
2KO
WT
B
MZM
MMM

Supplement: Additional file 2: Figure S2. — Marginal metallophilic macrophage (MMM) and marginal zone macrophage (MZM) in Ziz2 KO mice (A) Splenic sections from 9–10 weeks old female mice were stained with anti CD169 (Green) antibody. From three to four sections per mouse (one WT mouse and two KO mice were used) were stained and captured multiple area for localization of CD169+ cells. Dense CD169+ cells that are observed around MZ in WT sample (indicated by red line) seemed sparse in the KO sample (indicated by red-separated lines). Scale bars: 10 μm (B) FACS analysis for MZM and MMM. MZM or MMM were analyzed by staining splenocytes with anti SIGNR1 or anti CD169 antibody, respectively. There is no significant difference between the groups. WT: wild type. 2KO: Ziz2 KO. N = 9–18. [file 12979_2015_28_MOESM2_ESM.pptx]

## Slide 1
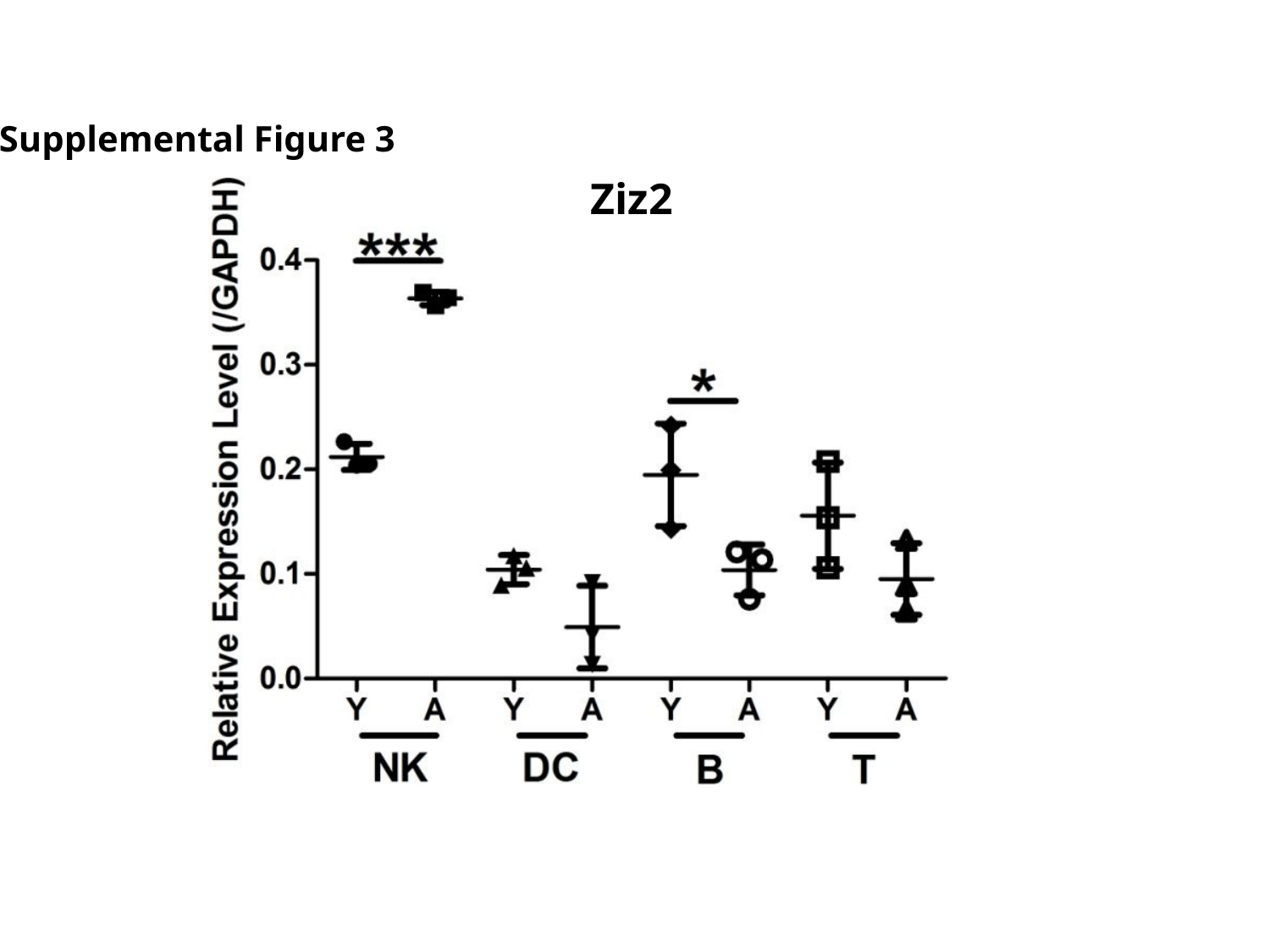

Supplemental Figure 3
Ziz2

Supplement: Additional file 3: Figure S3. — Ziz2 expression levels in splenic B/DC/NK/T cells from young (7–9 weeks old) or aged (2 years old) female mice. Splenic B, DC (dendritic cell), NK (natural killer), or T cells were sorted as B220+, CD11c+, NK1.1+, or CD3+ cells, respectively. Sorted cells were washed by PBS then immersed by RNA later (Ambion). RNA was extracted from the cells (that were pooled for three mice per group) by using QIAshredder and RNeasy Micro Kit (Qiagen). Reverse transcription was performed by using ReverTra Ace qPCR RT Master Mix with gDNA Remover (Toyobo). Five nano gram per well of cDNA was mixed with Thunderbird qPCR Mix (Toyobo) and TaqMan primer and prove (Assay ID: Mm01297557_m1 for Zizimin2 and Mm99999915_g1 for GAPDH as internal control, Applied Biosystems) then analyzed by PikoReal 96 Real-Time PCR System (Thermo scientific). Relative expression level of Zizimin2 to GAPDH are shown (triplicate of one representative experiment). The expression level of Zizimin2 in B cell was reduced in aged mice. *: P < 0.05 ***: P < 0.0001 N = 3 (triplicate). [file 12979_2015_28_MOESM3_ESM.pptx]
